# Diagnosis of Noonan syndrome and related disorders using target next generation sequencing

**DOI:** 10.1186/1471-2350-15-14

**Published:** 2014-01-23

**Authors:** Francesca Romana Lepri, Rossana Scavelli, Maria Cristina Digilio, Maria Gnazzo, Simona Grotta, Maria Lisa Dentici, Elisa Pisaneschi, Pietro Sirleto, Rossella Capolino, Anwar Baban, Serena Russo, Tiziana Franchin, Adriano Angioni, Bruno Dallapiccola

**Affiliations:** 1 Cytogenetics, Medical Genetics and Pediatric Cardiology, Bambino Gesù Children Hospital, IRCCS, Rome, Italy; 2 Illumina, Inc., San Diego, CA 92122, USA

**Keywords:** Noonan syndrome, Next generation sequencing, Molecular diagnosis, RASopathies

## Abstract

**Background:**

Noonan syndrome is an autosomal dominant developmental disorder with a high phenotypic variability, which shares clinical features with other rare conditions, including LEOPARD syndrome, cardiofaciocutaneous syndrome, Noonan-like syndrome with loose anagen hair, and Costello syndrome. This group of related disorders, so-called RASopathies, is caused by germline mutations in distinct genes encoding for components of the RAS-MAPK signalling pathway. Due to high number of genes associated with these disorders, standard diagnostic testing requires expensive and time consuming approaches using Sanger sequencing. In this study we show how targeted Next Generation Sequencing (NGS) technique can enable accurate, faster and cost-effective diagnosis of RASopathies.

**Methods:**

In this study we used a validation set of 10 patients (6 positive controls previously characterized by Sanger-sequencing and 4 negative controls) to assess the analytical sensitivity and specificity of the targeted NGS. As second step, a training set of 80 enrolled patients with a clinical suspect of RASopathies has been tested. Targeted NGS has been successfully applied over 92% of the regions of interest, including exons for the following genes: *PTPN11*, *SOS1*, *RAF1*, *BRAF*, *HRAS*, *KRAS*, *NRAS*, *SHOC*, *MAP2K1*, *MAP2K2*, *CBL*.

**Results:**

All expected variants in patients belonging to the validation set have been identified by targeted NGS providing a detection rate of 100%. Furthermore, all the newly detected mutations in patients from the training set have been confirmed by Sanger sequencing. Absence of any false negative event has been excluded by testing some of the negative patients, randomly selected, with Sanger sequencing.

**Conclusion:**

Here we show how molecular testing of RASopathies by targeted NGS could allow an early and accurate diagnosis for all enrolled patients, enabling a prompt diagnosis especially for those patients with mild, non-specific or atypical features, in whom the detection of the causative mutation usually requires prolonged diagnostic timings when using standard routine. This approach strongly improved genetic counselling and clinical management.

## Background

Noonan syndrome (NS, OMIM 163950) is an autosomal dominant developmental disorder [1] with a prevalence ranging between 1:1.000 and 1:2.500 live births [2]. This disorder is characterized by wide phenotype variability and shares some clinical features, as facial dysmorphisms, congenital heart defect (CHD), postnatal growth retardation, ectodermal and skeletal defects, and variable cognitive deficits [1,2], with other rare conditions, including LEOPARD syndrome (LS, OMIM 151100) [3], cardiofaciocutaneous syndrome (CFCS, OMIM 115150) [4], Noonan-like syndrome with loose anagen hair (NS/LAH, OMIM 607721) [5], and Costello syndrome (CS, OMIM 218040) [6]. This group of related disorders is caused by germline mutations in distinct genes, encoding for components of the RAS-MAPK signalling pathway. Based on common pathogenetic mechanisms and clinical overlap, these diseases have been grouped into a single family, the so-called neuro-cardio-facial-cutaneous syndromes (NCFCS), recently coined RASopathies [7,8]. NS is associated with *PTPN11*, *SOS1*, *KRAS*, *NRAS*, *RAF1*, *BRAF*, *SHOC2*, *MEK1* and *CBL* gene mutations [9-19], LS with *PTPN11, RAF1* and *BRAF* gene mutations [13,17,20,21], NS/LAH with *SHOC2* gene mutations [22], CFCS with *KRAS*, *BRAF*, *MEK1* and *MEK2* gene mutations [23,24], CS with *HRAS* gene mutations [25].

So far, the molecular characterization can be reached in approximately the 75-90% of affected individuals. Some distinct phenotypes are emerged in association with definite gene mutations.

Nowadays, due to high genetic heterogeneity of these disorders, which affect genes that all together span about 30 kb of genomic DNA, the standard diagnostic testing protocol requires a multi-step approach, using Sanger sequencing. The selection of the genes to investigate on a first diagnostic level depends on the frequency of their association with this disorder and their relationship with a distinct phenotype. For this reason, accurate clinical evaluation and close interaction between clinical and molecular geneticists are mandatory for selecting the genes to be first studied. By using this approach, the causative mutations can be identified in most of the cases. Some mutations cannot be identified during the first screening level since some phenotypes may be related to mutations in different causative genes or some clinical features associated with NS related disorders may not be evident at younger ages, or some extremely rare mutations are not routinely screened at first analysis. To detect these mutations, an additional screening level is required with a second panel of genes, which again should be guided by clinical geneticist. In these latter cases the molecular diagnosis requires a longer time before identifying the pathogenic mutation. Moreover, standard Sanger sequencing for multiple genes is also an expensive technique. Based on these notions, genetically heterogeneous disorders demand innovative diagnostic protocols, in order to be able to identify disease-causing mutations in a rapid and routinely way.

Here we report our personal experience on the use of targeted Next Generation Sequencing (NGS) for diagnosis of RASopathies. Our study suggests that this protocol can be easily used as a standard diagnostic tool to identify disease-causing mutations, with a straightforward workflow from genomic DNA up to genomic variants identification.

## Methods

### Subjects

Between June 2012 and June 2013, 80 patients (35 males and 45 females) with a clinical suspect of any RASopathy were consecutively enrolled in this study. Mean age was 8 years (range 2 months - 16 years). All patients had complete physical examination for major and minor anomalies by trained clinical geneticists (MCD, BD, RC). Two-dimensional Color-Doppler echocardiography, renal ultrasonography, and neurological/neuropsychiatric assessment for developmental delay or cognitive impairment were routinely performed. Clinical inclusion criteria were facial anomalies suggestive for RASopathies (presence of six or more features among hypertelorism, downslanting palpebral fissures, epicanthal folds, short broad nose, deeply grooved philtrum, high wide peaks of the vermilion, micrognathia, low-set and/or posteriorly angulated ears with thick helices, and low posterior hairline) [26], associated with almost one of the following clinical features: short stature, organ malformation (congenital heart defect or renal anomaly), developmental delay or cognitive deficit. All patients had normal standard chromosome analysis and array-CGH at a resolution of 75 kb.

A total of 10 DNA samples including 6 positive controls and 4 negative controls, previously characterized by standard Sanger sequencing were used as a validation set for establishing the amplicon resequencing workflow and assessing the analytical sensitivity and specificity of the targeted NGS. A second group of 80 DNA samples, extracted from patients manifesting the RASopathies phenotype, was used as training set. The patient’s genomic DNA was extracted from circulating leukocytes according to standard procedures and quantified with fluorescence-based method. Informed consent was obtained from the patients’ parents. The study was approved by the institutional scientific board of Bambino Gesù Children Hospital and was conducted in accordance with the Helsinki Declaration.

### Targeted resequencing

Targeted resequencing was performed using a uniquely customized design: TruSeq® Custom Amplicon (Illumina, San Diego, CA) with the MiSeq® sequencing platform (Illumina, San Diego, CA). TruSeq Custom Amplicon (TSCA) is a fully integrated DNA-to-data solution, including online probe design and ordering through the Illumina website sequencing assay automated data analysis and offline software for reviewing results.

### Probe design

**Figure 1:**
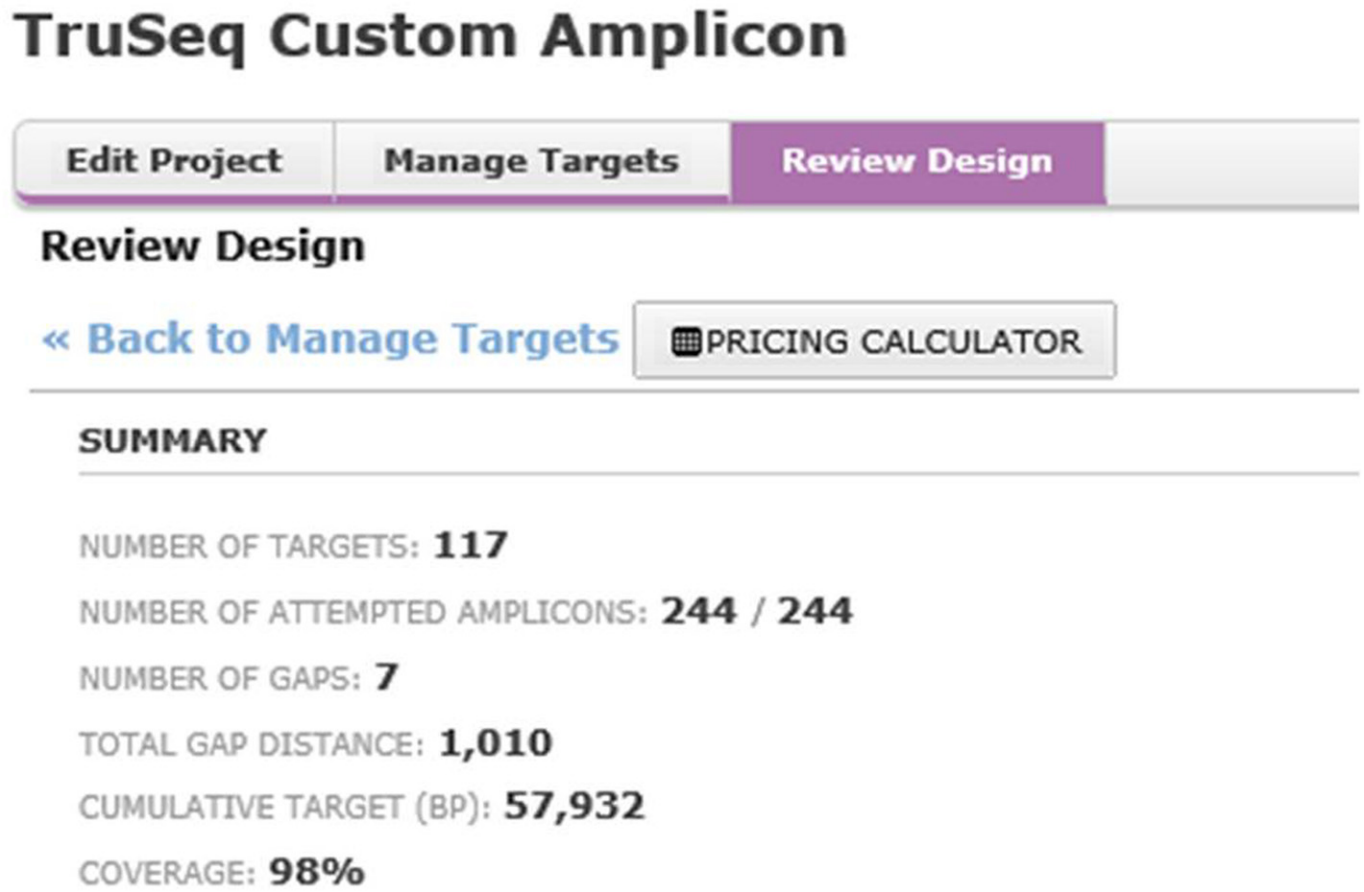
Screenshots of the designed panel within DS software.

Online probe design was performed by entering target genomic regions into Design Studio (DS) software (Illumina, San Diego, CA). Probe design (Locus Specific Oligos) was automatically performed by DS using a proprietary algorithm that considers a range of factors, including GC content, specificity, probe interaction and coverage. Once the design was completed, a list of 500 bp candidate amplicons (short regions of amplified DNA) was generated and the quality of each amplicon design assessed based on the predicted success score provided by DS. For some targets, when required, DS has been used by the operator to edit and improve the predicted success score to a minimum value of 60%. All exons with a lower success score have been removed from the design and excluded from the final TSCA panel. The design was performed over a cumulative target region of 57,932 bp and generated a panel of 244 amplicons with a coverage of 98% of the cumulative region (Figure 1). The choice of genes investigated in this panel has been made based on scientific evidence for a causative role in the disease [9-25]. The list of the 11 genes, for a total of 132 exons, is reported in Table 1.

**Table 1:** List of genes analyzed in this study and coverage percentage of the investigated exons

**Gene**	***PTPN11***	***SOS1***	***BRAF***	***RAF1***	***KRAS***	***NRAS***	***HRAS***	***SHOC2***	***MAP2K1***	***MAP2K2***	***CBL***
**Number of exons uploaded into DS**	15	23	18	16	5	4	5	8	11	11	16
**Number of exons entirely covered by DS with predicted success score >60%**	14	23	18	16	5	3	5	8	11	11	16
**Total exons covered by DS/ total exons uploaded into DS (%)**	98.5										
**Number of exons successfully sequenced with coverage > 30**	13	22	16	16	5	3	5	8	9	9	14
**Total exons successfully sequenced/ total exons covered by DS (%)**	92.4%										

### Library preparation and sequencing

TSCA kit generates desired targeted amplicons with the necessary sequencing adapter and indices for sequencing on the MiSeq® system without any additional processing. Library preparation and sequencing runs have been performed according to manufacturer’s procedure.

### Data analysis

The MiSeq® system provides fully integrated on-instrument data analysis software. MiSeq Reporter software performs secondary analysis on the base calls and Phred-like quality score (Qscore) generated by Real Time Analysis software (RTA) during the sequencing run. The TSCA workflow in Miseq Reporter evaluates short regions of amplified DNA (amplicons) for variants through the alignment of reads against a “manifest file” specified while starting the sequencing run. The manifest file is provided by Illumina and contains all the information on the custom assay. The TSCA workflow requires the reference genome specified in the manifest file (Homo sapiens, hg19, build 37.2). The reference genome provides variant annotations and sets the chromosome sizes in the BAM file output. The TSCA workflow performs demultiplexing of indexed reads, generates FASTQ files, aligns reads to a reference, identifies variants, and writes output files to the Alignment folder. SNPs and short indels are identified using the Genome Analysis Toolkit (GATK), by default. GATK calls raw variants for each sample, analyzes variants against known variants, and then calculates a false discovery rate for each variant. Variants are flagged as homozygous (1/1) or heterozygous (0/1) in the Variant Call File sample column. Because a SNP database (dbSNP (http://www.ncbi.nlm.nih.gov/projects/SNP) is available in the Annotation subfolder of the reference genome folder, any known SNPs or indels are flagged in the VCF output file. A reference gene database is available in the Annotation subfolder of the reference genome folder and any SNPs or indels that occur within known genes are annotated.

Each single variant reported in the VCF output file has been evaluated for the coverage and the Qscore and visualized via Integrative Genome Viewer (IGV) [27,28]. Based on the guidelines of the American College of Medical Genetics and Genomics [29], all regions that have been sequenced with a sequencing depth <30 have been considered not suitable for analysis. Furthermore we established a minimum threshold in Qscore of 30 (base call accuracy of 99.9%).

### Sanger sequencing validation

**Figure 2:**
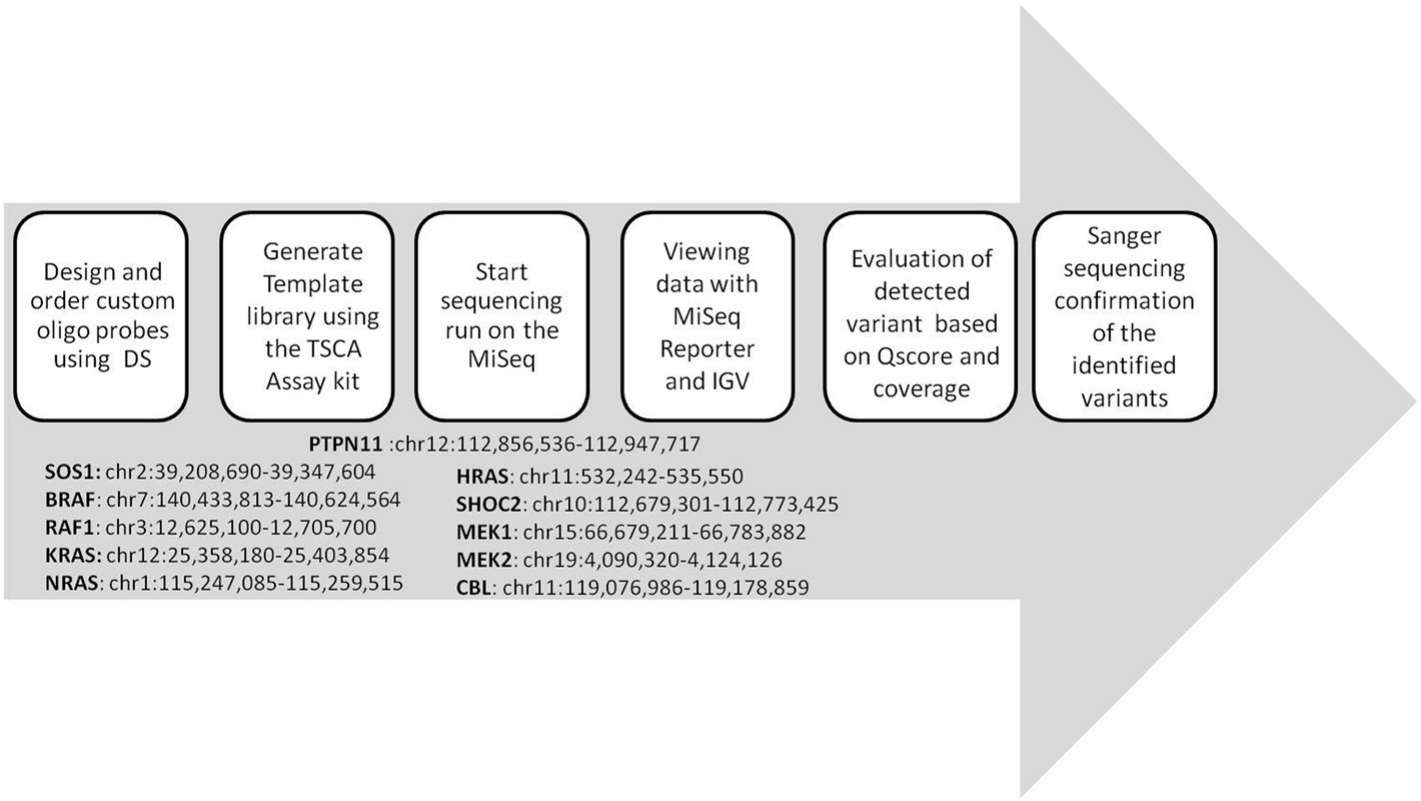
Flowchart of how the analysis was carried out.

All mutations identified by Miseq Reporter have been validated by Sanger sequencing using standard protocols and, where possible, family members were tested to detect the “*de novo*” origin of the mutation. Figure 2 shows the flowchart of the above described method.

## Results

### TSCA performance

All coding regions for genes reported in Table 1 have been uploaded into DS for a total of 132 exons (cumulative target region of 57,932 bp). The 98.5% of the exons uploaded were covered by the amplicon design, with a predicted success score ≥60%. The remaining exons not entirely covered by DS or with a predicted success score <60% have been excluded from final TSCA content panel. TSCA sequencing runs generated 120 exons successfully and steadily sequenced (sequencing depth >30, Qscore >30), providing a total coverage of 91% of the overall of the exons uploaded into DS, and a coverage of 92% when referring to the number of exons covered by DS (Table 1). The TSCA approach reduced up to 12 the number of exons requiring the standard Sanger sequencing analysis.

### Validation set

TSCA sequencing of 4 negative control confirmed the absence of any variant and the analysis of 6 positive control samples confirmed both the expected mutations and the allele state. All variants were identified with a mean coverage of 318 and a mean Qscore = 38, providing a detection rate of 100% for the validation set (Table 2). Both positive and negative control samples did not highlight any further unexpected variant, confirming the absence of any unreported variant in the validation set, and of any false positive result.

**Table 2:** List of patients with known mutations included in the validation set

**Patient ID**	**Gene**	**Mutation**	**Allele state**	**Mutation detected by TSCA sequencing**	**Coverage**	**Qscore**
**1**	*PTPN11*	Y63C	het	Y63C	374	38
**2**	*PTPN11*	N308D	het	N308D	519	39
**3**	*PTPN11*	T468M	het	T468M	525	40
**4**	*SOS1*	M279R	het	M279R	390	39
**5**	*SOS1*	I733N	het	I733N	78	37
**6**	*HRAS*	G12A	het	G12A	20	37

### Training set

Samples from training set were investigated in three different sequencing runs, with an average coverage of 200x, as set with DS. Among the patients, 38 mutations were identified in 6 of the 11 RAS pathway genes analyzed, *PTPN11* (22/38 = 58%), *SOS1* (9/38 = 23%), *BRAF* (2/38 = 5%), *MEK2* (2/38 = 5%), *RAF1* (2/38 = 5%), *CBL* (1/38 = 3%). The 38 variants identified from Miseq Reporter had an average coverage of 595x and an average Qscore of 38 (Table 3).

**Table 3:** Mutations identified by TSCA sequencing in patients enrolled in the training set

**Case**	**Phenotype**	**Gene**	**Mutation**	**Protein substitution**	**Allele state**	**Variant frequency**	**Coverage**	**Qscore**	**Reference**
**1**	NS	*PTPN11*	c.184 T > G	Y62D	het	0.448	460	39	[30]
**2**	NS	*PTPN11*	c.188A > G	Y63C	het	0.521	190	39	[9]
**3**	NS	*PTPN11*	c.188A > G	Y63C	het	0.54	512	39	[9]
**4**	NS	*PTPN11*	c.188A > G	Y63C	het	0.481	1046	38	[9]
**5**	NS	*PTPN11*	c.317A > C	D106A	het	0.495	632	39	[31]
**6**	NS	*PTPN11*	c.328G > A	E110K	het	0.486	702	36	[31]
**7**	NS	*PTPN11*	c.417 G > C	E139D	het	0.498	406	38	[30]
**8**	NS	*PTPN11*	c.661A > G	I221V	het	0.482	737	39	*p.s*
**9**	NS	*PTPN11*	c.767A > G	Q256R	het	0.514	290	36	*p.s*
**10**	NS	*PTPN11*	c.854 T > C	F285S	het	0.406	64	39	[30]
**11**	NS	*PTPN11*	c.922 A > G	N308D	het	0.526	812	38	[9]
**12**	NS	*PTPN11*	c.922 A > G	N308D	het	0.508	1174	39	[9]
**13**	NS	*PTPN11*	c.922 A > G	N308D	het	0.505	3126	39	[9]
**14**	NS	*PTPN11*	c.922 A > G	N308D	het	0.486	3111	39	[9]
**15**	NS	*PTPN11*	c.923 A > G	N308S	het	0.555	119	40	[30]
**16**	NS	*PTPN11*	c.1183G > T	D395Y	het	0.556	561	38	*p.s*
**16**	NS	*PTPN11*	c.1186 T > C	Y396H	het	0.557	560	37	*p.s*
**17**	NS	*PTPN11*	c.1226G > C	G409A	het	0.444	178	38	[32]
**18**	NS	*PTPN11*	c.1282G > T	V428L	het	0.502	416	38	*p.s*
**19**	LS	*PTPN11*	c.1403C > T	T468M	het	0.467	319	40	[20]
**20**	LS	*PTPN11*	c.1492 C > T	R498W	het	0.573	185	35	[33]
**21**	LS	*PTPN11*	c.1492 C > T	R498W	het	0.521	142	38	[33]
**22**	NS	*SOS1*	c.755 T > C	I252T	het	0.528	212	39	[34]
**23**	NS	*SOS1*	c.806 T > G	M269R	het	0.564	140	38	[34]
**24**	NS	*SOS1*	c.806 T > G	M269R	het	0.496	391	38	[34]
**25**	NS	*SOS1*	c.1310 T > A	I437N	het	0.46	302	40	[34]
**26**	NS	*SOS1*	c.1649 T > C	L550P	het	0.516	275	39	[34]
**27**	NS	*SOS1*	c.1649 T > C	L550P	het	0.428	428	39	[34]
**28**	NS	*SOS1*	c.2104 T > C	Y702H	het	0.52	421	37	[34]
**29**	NS	*SOS1*	c.2371C > A	L791I	het	0.576	363	37	*p.s*
**30**	NS	*SOS1*	c.2371C > A	L791I	het	0.546	108	39	*p.s*
**31**	NS/CFCS	*BRAF*	c.1694A > G	D565G	het	0.463	341	39	*p.s*
**32**	CFCS	*BRAF*	c.1802A > T	K601I	het	0.538	1120	37	[17]
**33**	CFC	*MEK2*	c.326C > T	A110 T	het	0.505	299	37	*p.s*
**34**	CFC	*MEK2*	c. 395 T > G	G132D	het	0.533	227	38	[35]
**35**	NS	*RAF1*	c.785 A > T	N262I	het	0.504	135	39	*p.s*
**36**	NS	*RAF1*	c.781C > T	P261S	het	0.524	143	39	[13]
**37**	NS	*CBL*	c.2350G > A	V784M	het	0.428	173	36	*p.s*

p.s: present study.

**Figure 3:**
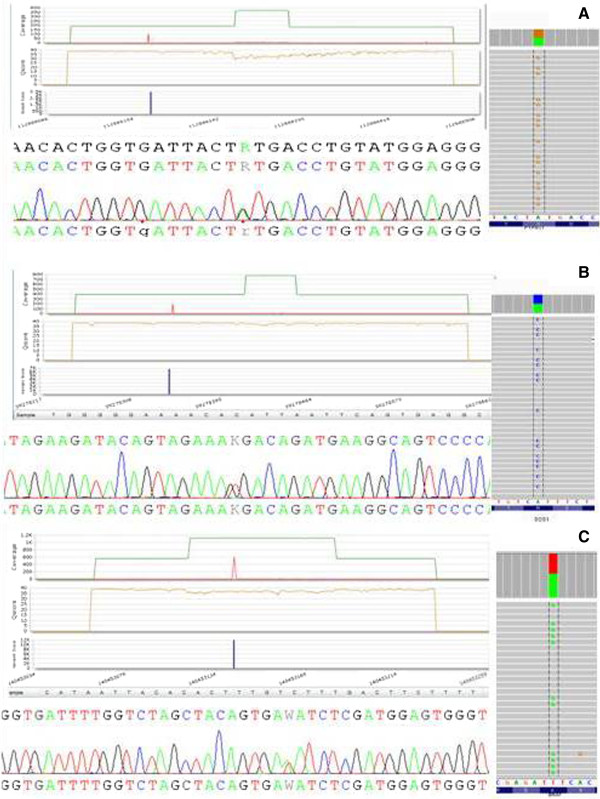
**An example of three different mutations (A. *PTPN11*:Y63C; B. *SOS1*: M269R; C. *BRAF*: K601I) identified by Miseq.**

All variants have been confirmed by Sanger sequencing and IGV, indicating the absence of any false positive result in the training set group (Figure 3). Moreover, to exclude any possible false negative event, 10 negative samples randomly selected, have been further analyzed by Sanger sequencing (only “hot spots” exons) and 30 additional samples have been analyzed for *PTPN11*, using NGS and Sanger sequencing and all of them provided negative results.

### Reproducibility

**Figure 4:**
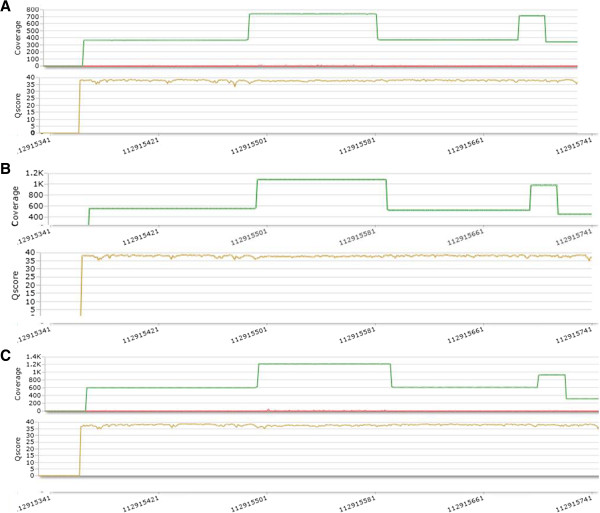
Performance of the same target (PTPN11_exon8) region through 3 different sequencing runs (A. first run; B. second run; C. third run).

TSCA sequencing showed 100% reproducibility for all 120 exons, independently from DNA samples and sequencing runs, making this approach compatible with a diagnostic purpose. Figure 4 illustrates the performance of the same target region through three sequencing runs.

## Discussion

The term RASopathy applies to a group of genetic disorders characterized by similar phenotypes, caused by mutations in the RAS MAPK pathway. These phenotypes are characterized by a high degree of genetic heterogeneity, since individual diseases can arise from mutations in different genes. In addition, since different RASopathies share similar clinical features, their molecular characterization is complex, time consuming and expensive.

In order to improve the molecular testing of RASopathies, in this study we investigated a protocol based on a targeted NGS using MiSeq Illumina platform enabling the analysis of all known causative genes in up to 96 patients in a single sequencing run. In particular, we analyzed 80 patients and identified 38 mutations in 6 of 11 RAS-pathway genes, including *PTPN11* (22/38 = 58%), *SOS1* (9/38 = 23%), *BRAF* (2/38 = 5%), *MEK2* (2/38 = 5%), *RAF1* (2/38 = 5%), *CBL* (1/38 = 3%). The relative frequency of mutations in the tested genes was in agreement with published results [30,36,37]. As shown in Table 3, in many patients the causal mutation was identified in the gene considered the most suitable candidate, based on frequency of mutations and the phenotypic characteristics. As expected, most NS patients had a *PTPN11* mutation, while the second most frequently mutated gene was *SOS1,* followed by *RAF1*. Three patients with LEOPARD syndrome had one of the *PTPN11* recurrent mutation previously associated with this phenotype (T468M or R498W) [20,33]. Among patients heterozygous for a pathogenic *BRAF* mutation, one was clinically diagnosed as being affected by CFCS, while in the other case clinical evaluation was unable to conclude whether he was affected by NS or CFCS. The patients’ age at diagnosis is obviously important: this latter subject was 2 year-old at time of clinical evaluation, when he displayed only some features of CFCS [38]. Two other patients with CFCS had a mutation in *MEK2* gene, which is less commonly mutated in this disorder*.* In addition, one NS patient had a mutation in *CBL,* a gene rarely associated with this disorder. Since all genes have been analyzed in one run, the present protocol allowed to reach the simultaneous identification of mutations affecting both the most frequent and rare genes, with a significantly reduction of time needed to reach the molecular characterization of the patient. This point is important for early diagnosis and classification of the different RASopathies, allowing a more appropriate management and counseling.

A wide spectrum of *PTPN11* mutations has been associated with NS, including some rare mutations: I221V, Q256R, F285S, V428L (Table 4). All these variants are associated with the distinct facial gestalt of NS, which was markedly expressed in one patient (case no.10) with F285S and mildly expressed in the others. Familial transmission from an affected individual has been found in one instance, and suspected in another case (no.8), where the affected proband’s brother was referred to have membranous subaortic stenosis and cryptorchidism. Mental retardation or cognitive deficit was not associated with these mutations, with the exception of F285S mutation. Interestingly, the patient heterozygous for this latter mutation had a congenital pancreatic cyst, an unusual malformation in the RASopathies. In one patient (case no.16) we identified two unpublished mutations affecting two consecutive *PTPN11* aminoacids, D395Y and Y396H. Both mutations were inherited from the affected father. Variability of clinical expression in this family was recognizable, since facial anomalies of NS were associated with developmental delay in the son only. The father had congenital total alopecia as distinctive feature. It is evident that the phenotype related to these mutations is quite atypical, showing common Noonan-like facial anomalies associated with variable additional neural and ectodermal features.

**Table 4:** **Clinical features of the patients carrying rare *PTPN11 *** **mutations**

	**Case n°8**	**Case n°9**	**Case n°10**	**Case n°16**	**Father case n°16**	**Case n°18**	**Mother case n°18**
**Sex**							
**Short stature**							
**Macrocephaly**	+	-	+	-	-		
**Hypertelorism**							
**Downslanting palpebral fissures**							
**Palpebral ptosis**	-	-	+	-	+	+	+
**Epicanthal folds**	+	+	-	+	-		
**Short broad nose**	+	+	-	+	-	-	
**Deeply grooved philtrum**	+	+	+	+	+		
**High wide peaks of the vermilion**	+	+	+	+	+	+	+
**Micrognathia**	-	+	-				
**Low-set and/or posteriorly angulated ears with thick helices**							
**Low posterior hairline**	-	+	+	-	-	+	-
**Thorax anomalies**	-	+	+	-	-	+	-
**Cardiac defect**	+	+	-	-	-		
**- PVS**	-	+	+	-	-	-	-
**- ASD**	-	-	-				
**- VSD**	-	-	-	-	-		
**- PDA**	-	+	-	-	-		
**Arrhythmia**	-	-	-	-	-	WPW	-
**Renal anomaly**	-	-	-	-	-	-	-
**Cryptorchidism**	NA	-	-	+	NA		
**Developmental delay or cognitive deficit**							
**Alopecia**	-	-	+	+	-		
**Pancreatic cyst**	+	-	-	-	-		
**Angioma**	-	-	-	-	-		
**Inheritance**	NT	pat	NT	mat	NT		

ASD, atrial septal defect; VSD, ventricular septal defect; HCM, hypertrophic cardiomyopathy; mat, maternal; NA, not applicable; NT, not tested; pat, paternal; PDA, patent ductus arteriosus; PVS, pulmonary valve stenosis; WPW, Wolf-Parkinson-White.

+present; -not present.

The other 43 patients enrolled in this study were negative for the investigated RAS genes. The proportion of negative patients was higher compared to previous reports, likely because clinical inclusion criteria were less stringent compared to other studies [39-41], being based on NS facial anomalies and almost only one additional feature. Interestingly, the mother of patient n°18 was included in this study being the mutated parent of an affected child, although the minimal clinical criteria for diagnosis of NS were not present.

All the variations have been confirmed by Sanger sequencing as well as the 4 negative control patients. These data indicated a 100% detection rate of mutations involved in RASopathies and, most important, all these results have been obtained with MiSeq on board analysis, being the bioinformatics examination performed only adopting the user friendly Miseq Reporter software.

The absence of any false negative and false positive results and the possibility to have an easy and accurate data analysis make this approach a good diagnostic tool. Furthermore, the library preparation workflow is easy and the TSCA kit performance is stable. In fact, all different experiments for the RAS pathway genes in each run provided the same results in terms of coverage and quality (Figure 4).

**Figure 5:**
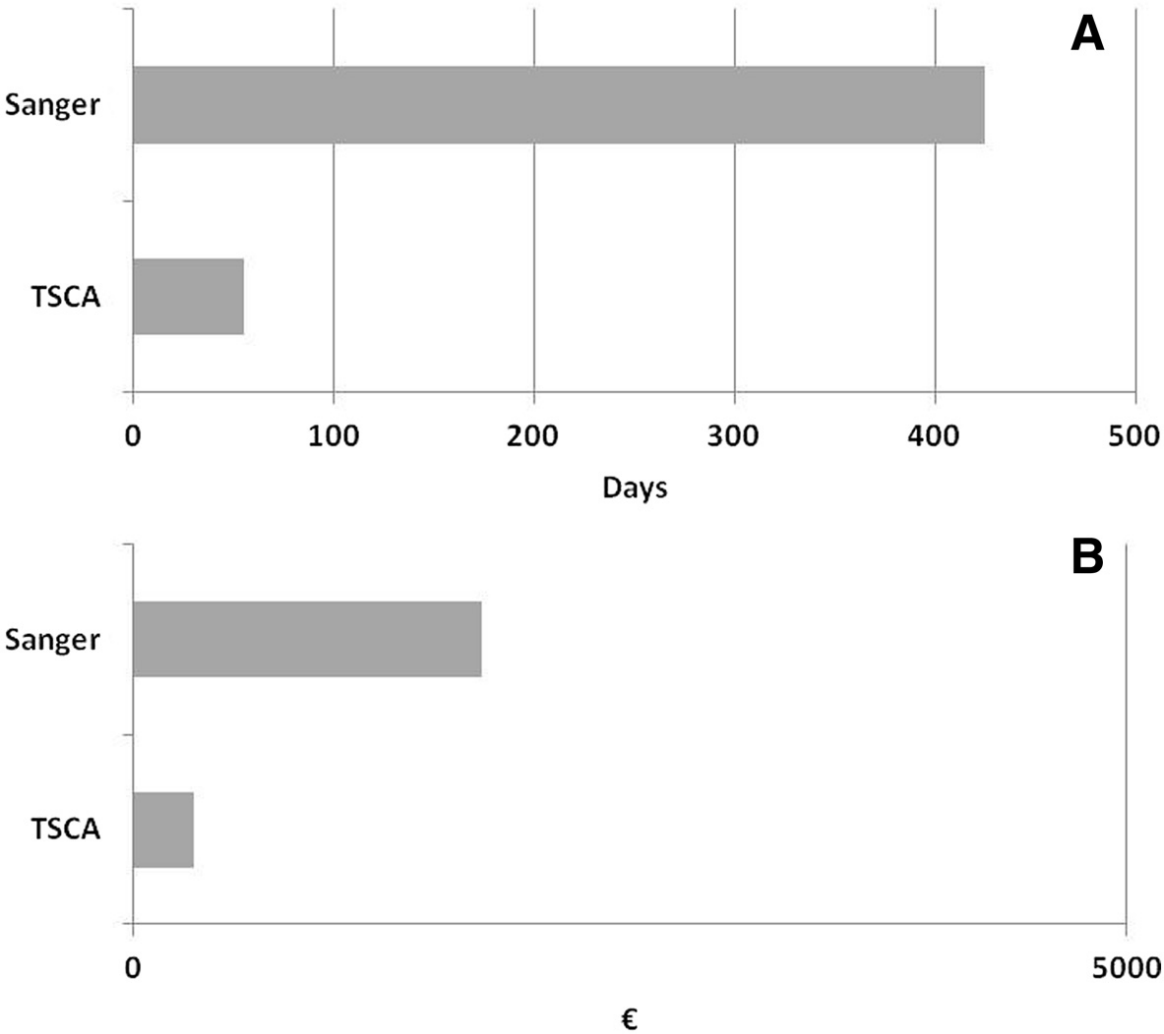
Comparison of time (A) and cost (B) between NGS and Sanger sequencing.

Two major points to be considered in the diagnostic protocols include the time needed to complete the entire workflow and the costs that this approach requires. Targeted NGS analysis of the complete coding sequences of the 11 genes in the RAS pathway for 96 patients takes about two months, including ten days for library preparation and data analysis and about 45 days to characterize uncovered regions using Sanger sequencing. Conversely, the use of Sanger sequencing to analyze the full coding sequence of the 11 genes would take about 16-18 months for the same number of patients. We also calculated that the cost of NGS analysis applied to the 92% of the regions of interest, plus Sanger sequencing of for the remaining regions, would cost 6 time less than the cost of a protocol entirely based on Sanger sequencing (Figure 5). However, time and cost could be further reduced, by designing a new panel which includes also *RIT1*, a gene recently associated to NS [42], resulting in a 100% coverage of the cumulative region. This result likely makes the Sanger sequencing irrelevant for the analysis, further reducing the time and the cost of the entire process.

## Conclusion

This study demonstrates that NGS can be successfully applied to the molecular testing of RASophaties with a remarkable gain of time and less cost, while maintaining the high quality of the results. Consistent with available records, our data confirm that the genetic mechanism underlying the RASopathies is due to germline mutations in different genes encoding for components of the RAS-MAPK signalling mutations, with *PTPN11*, followed by *SOS1*, being the most frequently mutated genes in our cohort. Moreover, the use of NGS protocol has allowed, with a high standard in terms of coverage and quality, an early detection of rare mutations in other RAS-MAPK genes, avoiding the use of standard Sanger sequencing approach and the related enlarged cost and time consuming issues. Taken all together, these data highlight the usefulness of a molecular characterization that lead to an early diagnosis especially for patients with mild, nonspecific or atypical features and might direct to a more appropriate genetic counselling and clinical management.

## Abbreviations

NS: Noonan syndrome; LS: LEOPARD syndrome; CFCS: Cardiofaciocutaneous syndrome; NS/LAH: Noonan-like syndrome with loose anagen hair; CS: Costello syndrome; NGS: Next generation sequencing; M.C.D: Maria Cristina Digilio; B.D: Bruno Dallapiccola; R.C: Rossella Capolino; TSCA: TruSeq custom amplicon; DS: Design studio software; GATK: Genome analysis Toolkit; RTA: Real time analysis software; IGV: Integrative genome viewer; p.s.: Present study.

## Competing interests

The authors declare that they have no competing interests.

## Authors’ contributions

FRL: carried out the molecular genetic studies, the analysis and interpretation of data and drafted the manuscript. RS: participated to analysis and interpretation of data and drafted the manuscript. MCD, BD and MLD performed the clinical examination of the patients and have been involved in drafting the manuscript. MG, SG, EP, PS and SR: contribute to perform the molecular genetic studies and analysis and interpretation of data. RC and AB: performed the clinical examination. TF: contribute to perform the analysis and interpretation of data. AA: contribute to concept the study and has been involved in drafting the manuscript. All authors read and approved the final manuscript.

## Pre-publication history

The pre-publication history for this paper can be accessed here:

http://www.biomedcentral.com/1471-2350/15/14/prepub
